# Reversible Photoswitching of Carbon Dots

**DOI:** 10.1038/srep11423

**Published:** 2015-06-16

**Authors:** Syamantak Khan, Navneet Chandra Verma, Abhishek Gupta, Chayan Kanti Nandi

**Affiliations:** 1 School of Basic Sciences, Indian Institute of Technology Mandi, Himachal Pradesh, 175001, India

## Abstract

We present a method of reversible photoswitching in carbon nanodots with red emission. A mechanism of electron transfer is proposed. The cationic dark state, formed by the exposure of red light, is revived back to the bright state with the very short exposure of blue light. Additionally, the natural on-off state of carbon dot fluorescence was tuned using an electron acceptor molecule. Our observation can make the carbon dots as an excellent candidate for the super-resolution imaging of nanoscale biomolecules within the cell.

Single molecule imaging and super resolution microscopy in live cell down to nanometer scale by breaking the diffraction limited regime has been one of the biggest success stories in the past decades^1^,^2^,^3^, with the Nobel Prize in Chemistry for the year 2014. Photoswitchable fluorescent probes have been instrumental for their unique ability of photo-conversion between a bright fluorescent state and a dark state for hundreds of cycles^4^,^5^. The number of photons emitted per cycle determines the effective brightness of the molecule. Again the brightness of each cycle must have a relation with the maximum number of allowed cycles before permanent photobleaching. Given the fact that it is possible to detect thousands of photons from a single organic fluorophores before it permanently photobleaches, localization of individual fluorophores with up to nanometer level accuracy is feasible. All these important properties are currently being researched in conventional dyes, fluorescent proteins and quantum dots^4^,^6^,^7^,^8^,^9^. Most of the current super-resolution imaging methods (STORM, SPDM, PALM) rely on controlled photoswitching or photoactivation of fluorescent probes.

Here, we introduce the photoswitching of the red emissive carbon nanodots, which can show its potential in high resolution bioimaging. Though the origin of the fluorescence is not yet understood, the immense potential of these so-called carbon dots is irrefutable. This is due to their superior photostability, low toxicity, excellent biocompatibility, low cost and abundance of raw material in nature^10^,^11^. Mechanistically, the photoluminescence from carbon dots may be attributed to the presence of surface energy traps that become emissive upon surface passivation^12^,^13^. Nevertheless, all of these hypotheses are still debatable. The observed photoluminescence of carbon dots in ensemble experiment, in general, is the average effect of all the possible combinations of size, surface defects, charge and oxidation states, that arise from harsh synthetic methods and different precursor materials. Further, most of the reported carbon dots are exclusively bright blue emitters, while the red emission is very rare, suggesting that either the longer wavelength emissive particles (yellow/red) are very rare in the system or their emissive sites are not energetically stable. Nevertheless, whether it is from a single particle with multichromophoric groups or from different particles is still a topic of debate.

In our study, carbon dots were synthesized using the reported methods of microwave assisted carbonization of biocompatible chitosan molecule followed by surface passivation by poly ethylene glycol (PEG)^14^. The materials were characterized by the standard techniques (Figure S1). The presence of π-π* transition of the C-C structures along with aromaticity was indicated by two UV absorption peaks at 257 nm and 302 nm respectively. The ensemble measurements showed a long range multicolor fluorescence signal with a maximum at around 412 nm when excitation wavelength was varied from 280-600 nm. The fluorescence brightness and as well as the photostability of the red particles were greatly enhanced when microdrops were evaporated on the glass slide at ambient temperature. These radical changes of fluorescence properties reveals the strong interaction of emissive sites with its chemical micro environment. This initial observation indicated that the solvent environment doesn’t allow the red emissive sites to come into the play and somehow lock them into a non-fluorescent (dark) state. This intensity reversal of the red emission (from extremely low to very bright), when viewed an air drying drop-casted sample of carbon dots under confocal microscope, inspired us to explore its photoswitching properties (Figure S2, Video S1).

The photo decay of red emission was relatively faster with a quick drop in the observed photon count (Figure S3). However, interestingly, a number of particles were found to switch back to the bright fluorescence state after exposing them to a 401 nm laser for a very short time. Further exposer of 639 nm red laser again promoted these molecules into the dark state which was revived by another flash of 401 laser (Fig. 1, Video S2). Practically, a large number of cycles were observed, remarkably without any reduction of intensity after photoswitching. The fourth and sixth cycle, with a slightly higher exposure time of blue light, resulted a better recovery of fluorescence. This intriguing phenomenon of reversible photoswitching has been reported in very few molecules till date, though never in carbon dots. In fact, many of the known switchable dyes require UV irradiation, which limits their potential biological applications due to their high penetrating power^15^. Instances of photoswitching with the help of visible light are very rare^16^,^17^.

To get further insights into the mechanism and the effect of any chemical environment, as observed in dried droplets, we set out to measure the single molecule fluorescent fluctuations^18^ of these materials. Single fluorescing quantum systems almost invariably display discrete fluctuations of their fluorescence intensity with time, commonly known as blinking. Blinking is generally interpreted as arising from transitions of the fluorophore to a non-fluorescent/very less fluorescent state, known as a “dark” state, from which it return to the initial bright state after some time^19^,^20^. When the system does not return to the fluorescing state, the process is called bleaching, the permanent photochemical destruction. Molecules were trapped in a poly vinyl alcohol film in the presence of both electron donor (reducing environment) and electron acceptor (oxidizing environment) molecules. For this purpose ascorbic acid (electron donor) and methyl viologen (electron acceptor) were chosen, while no oxygen removal system was used as the presence of molecular oxygen in the solution has both its merits and demerits. It acts as an efficient triplet state quencher but enhances the photobleaching via photo-oxidation on the other hand.

Single molecule transient showed a single step bleaching in presence of ascorbic acid, while fluorescence fluctuation with a long lived dark state^19^,^20^ was observed in the presence of methyl viologen. (Fig. 2a,c). We ascribe the formation of a positive radical by an electron transfer process from the excited state (Fig. 2b). Ascorbic acid acts as an electron donor and rescues the molecules which are trapped in the radical (cation) dark state. Additionally, it inhibits the radical formation pathway in a competitive manner. This eventually aids the molecules to relax from their triplet state itself avoiding the dark state transition. But methyl viologen induces the dark state of the molecule by spontaneously taking up an electron form the excited state. Additionally, it inhibits the pathway by which the radical comes back to the ground state. Thus, methyl viologen basically ease this process by lowering the potential energy barrier of the electron transfer reaction (Fig. 2d). The reverse action of ascorbic acid is very intuitive. Interestingly, our proposed mechanism with methyl viologen immediately indicates a possibility of photoswitching (reverse reaction) as the fluorophores quickly enter into the dark state with a low potential energy barrier.

A decrease in the steady state fluorescence intensity in the presence of methyl viologen and an increase in the presence of ascorbic acid possibly accounts for the acceleration and retardation of a radical cation formation, respectively (Figure S4). So in summary, this radical blinking and entry into the dark state occurs via a photo-excited transition state and an electron acceptor lowers the energy barrier, easing this process while an electron donor raise it further (Fig. 2d). The higher energy barrier with ascorbic acid readily suggests that the molecule has a high chance of permanent photochemical destruction before it ever crosses the high energy barrier. Naturally, in methyl viologen, it has a minimum chance of the same as it never reaches that high energy state. Rather, a low energy transition state easily promotes the molecule towards the dark state. This must be the reason why multiple cycles of photoswitching hardly allows the permanent photo-damage. The cycle occurs every time through a very low energy maximum. This concept could be crucial for designing future photoswitchable fluorophores for their precise applications.

The same experiment was also performed using an EMCCD detection unit (iXon, Andor) with our home-built single molecule microscopy setup. A different laser, 532 nm was used this time to record the fluorescence fluctuation of the dots at a single molecule level. Video S3, detected in EMCCD, shows the same phenomena which were observed using photomultiplier assembly. Addition of 5 mM ascorbic acid caused an overall intensity increment and rapid photobleaching (single step bleaching) at the same time. Methyl viologen of the same concentration promoted a prolonged on-off switching (Dark state blinking) with a decreased brightness. Figure 3 shows single molecule traces calculated from the EMCCD detection with 100 ms integration time. Above 75% of the analyzed molecules showed those characteristic single molecule fluorescent traces.

Next, the carbon dots are known to possess a large number of energy traps (both emissive and non-emissive) on their surfaces. The high energy non-emissive traps which can effectively absorb the higher frequency of light might have a role at the core of photoswitching. As the chemical architecture and the origin of multicolour fluorescence of carbon-dots are not yet known, it is indeed difficult to accurately predict the origin of photoactivation.

Our experiment only confirms that electron transfer has a role in this process. A loss of electron disrupts the emission process which could be repaired by a higher energy laser. It is assumed that new sites are not created by photoactivation, rather the same emissive sites are reactivated back to the bright state. Creation of a different site is unlikely as the photoactivation was not observed in the presence of an electron donor, where molecules suffer single step permanent photobleaching. Now, electron transfers from the neighboring high energy traps can efficiently switch the chromophore back from the dark radical state to a bright fluorescent state (Fig. 4). This implies, there is also a small possibility of spontaneous electron transfer from different energy traps, even without any external light. In fact, this has been observed when we studied fluorescence recovery after photobleaching within a self-assembled monolayer of carbon dots.

The carbon dots were drop casted on the glass slide and allowed to dry. Here a concentration variation can give rise to various structures due to complex dynamics within the drop and this has been found to be true in most of the colloidal solution. For instance, a diluted drop can produce a nice coffee-ring at the drop boundary while a highly concentrated solution will result in a densely packed structure throughout the drop area^21^. However, a controlled experiment can produce self-assembled nanoparticle colony (Figure S5), which are believed to be patches of nanoparticle monolayer^22^. We observed a fast and spontaneous fluorescence recovery of the nanoparticle monolayer upon cessation of laser excitation. We used three different lasers (401, 488 and 639 nm) to detect three different emissions and a single point excitation was used to study photobleaching (Fig. 5, Figure S6). It is noteworthy that, the blue emission decays slowly and recovers very fast, while the red emission decays fast and recovers slowly. This spontaneous recovery of fluorescence, which is quite intriguing, suggests a possibility of mutual transfer of photo-excited electrons from the neighboring sites. Naturally mutual transfer will not be possible in the solution or in another environment where the molecules are well separated from each other. The increased hydrogen bonding in monolayers brings those molecules close enough for this electron transfer to take place.

In conclusion, the controllable photoswitching will add a lot of merits to the research field of carbon dots, making it a new possibility for the super resolution imaging and diffusion studies of the nanoscale biomolecules within the cell. However, it must be mentioned that there are number of questions which remain unanswered at this stage. In particular, the exact molecular mechanism of photoswitching can only be deciphered with the knowledge of chemical structure and physical origin of multicolor fluorescence which is still elusive. The electron transfer mechanism clearly has a major role in the process. But apart from surface state activation, possibilities like isomerism (e.g. cis-trans), protonation-deprotonation, disruption of conjugation system exist and any of them can practically promote a dark state. The role of 401 nm laser can also be very clearly understood, only when the dark state is chemically known. Hence, research efforts to chemically characterize carbon dots and to understand their origin of the fluorescence would be extremely fruitful.

## Methods

### Materials

All Glasswares were washed with aqua regia (3 HCl: 1 HNO_3_), followed by rinsing several times with double distilled water. Chitosan, Polyethylene glycol (PEG), ascorbic acid and methyl viologen were purchased from Sigma Aldrich. Double distilled 18.3 mΩ deionized water (Elga Purelab Ultra) was used throughout the preparation of solutions.

### Synthesis and purification of carbon dots

2% Chitosan (CHS) gel was prepared by dissolving 2 grams of CHS in 99 ml of water in the presence of 1 ml of acetic acid, under vigorous stirring condition. 25 ml of PEG was dissolved in 75 ml water (25% PEG solution). For synthesizing carbon dots, 4.5 ml of CHS gel was mixed with 4.5 ml of 25% PEG in a beaker. In the solution 1 ml of 5 M NaOH was mixed. This mixture was allowed to heat at 600 W power of microwave for 3 minutes. The resulting material is diluted up to 15 ml with distilled water and the solution was filtered by Whatman filter paper. Small volume of resulting solution (1.5 ml) was ultra-centrifuged (sorvall Lynx 6000, thermo scientific) at 23000 rpm for 30 minutes twice. The pellets were removed each time and the final supernatant was collected.

### UV-Vis and steady state fluorescence

The UV-Vis spectra of synthesized carbon dots were measured using Shimadzu UV-Vis 2450 spectrophotometer. The spectra were collected using a quartz cuvette with 10 mm path length. 1 ml of carbon dots solution was used to measure the spectra. The measurements were repeated three times. The Steady state fluorescence was measured using Horiba Fluorolog-3 spectrofluorometer. 1 ml of carbon dots solution was used to measure the spectra. Multicolor fluorescence spectra recorded from 280 to 600 nm with variable excitation with 20 nm increment.

### Transmission Electron Microscope (TEM)

The particle size and dispersity of the synthesized nanoparticles were performed using a TECNAI G2 200 kV TEM (FEI, Electron Optics) electron microscope with 200 kV input voltage. TEM grids were prepared by placing 5 μL diluted and well sonicated sample solution on a carbon coated copper grid followed by complete evaporation at room temperature. Precautions were taken to avoid contamination from various sources like dust particles and glasswares.

### Fourier Transform Infrared Spectroscopy (FTIR)

FTIR spectra of carbon dots were measured using a Perkin-Elmer FTIR spectrophotometer equipped with a horizontal attenuated total reflectance (ATR) accessory containing a zinc selenide crystal and operating at 4 cm^−1^ resolution. The use of the spectral subtraction provided reliable and reproducible results. KBr pellet has been used as a reference and for the baseline correction.

### Atomic Force Microscopy (AFM)

AFM analysis of the synthesized cabon dots were carried out using a Digital Instruments Bruker AFM for particle size determination. Standard Veeco tapping mode silicon probes were used for scanning the samples. Typically, aqueous suspensions of carbon dot samples were dried on silicon substrate for 3 h. Once dried, samples were placed on the AFM and scanned. Pertinent scanning parameters were as follows. Resonant frequency (probe): 60-80 kHz; Tip velocity: 4 μm/s for 2 μm, 15 μm/s for 5 μm and 30 μm/s for 10 μm; Aspect ratio: 1:1 and Resolution: 512 samples/line, 256 lines.

### Confocal Microscopy

The dried carbon dots were studied under the inverted confocal microscope (A1R+, Nikon, Japan) using a 1.4 NA oil-immersion 60X objective. Similar powers of 401, 488 and 639 nm lasers were used to excite the sample at room temperature (25 °C) using appropriate dichroic and filters in the optical path. The intensity profiles were analyzed within the region of interest (ROI) of the sample. Bright fluorescent spots including blue/green/red emission were observed in different samples. Some of the spots were multicolored while few were purely single colored. In most of the observations, the red emissive spots were found to decay quickly in comparison to the other colors.

### Single molecule transients, blinking and photoswitching Study

Carbon dots were immobilized on cleaned glass coverslips by trapping in poly vinyl alcohol (PVA) and spin coating at 3000 rpm. PVA solution (1 g/ml) was prepared in PBS buffer at pH 7.5. Microscope cover-slips were cleaned by immersing in NaOH (5.0 M) followed by ethanol and DI water. Same microscopic setup was used for the single molecular study with only 639 nm laser. The emission beam was directed through a side-port of the microscope delivering it to a Hybrid Photomultiplier Detector Assembly single photon counting module (PicoQuant GmBh. Berlin, Germany). For dual channel detection, it was separated by a F48-620 dichroic and further purified by two bandpass filters (690/70 and 480/35). The lasers 639 nm and 401 nm were alternately (manually) switched on and off to record the seven cycles of photoswitching, while photons were detected in the respective channels (blue/red). All data were analyzed using the SymPhoTime 64 software (PicoQuant GmBh).

EMCCD detection was performed using a home built single molecule set-up. A diode laser of 532 nm was used which was passed through a TIR objective (Nikon) before exciting the sample. Appropriate dichroic/filter sets were used. Signals were detected with an EMCCD camera (Andor, iXon Ultra) with 100 ms integration time. All data were analyzed using the Andor Solis software.

### Fluorescence recovery in carbon dot monolayer

To form the nanoparticle monolayer, variable concentrations of solutions were drop casted on glass slide and evaporated. All three lasers were focused on the predefined ROI point with an exposure time of 10 s. The images of fluorescence recovery were captured with a very low exposure time to avoid further photobleaching. The fluorescence recovery was analyzed by plotting the fluorescence intensity of the photobleached region. A circular region of interest (ROI) was constructed around the point AUX and the mean intensities were calculated to minimize the error.

## Additional Information

**How to cite this article**: Khan, S. *et al.* Reversible Photoswitching of Carbon Dots. *Sci. Rep.* **5**, 11423; doi: 10.1038/srep11423 (2015).

## Supplementary Material

Supplementary Information

Supplementary Video 1

Supplementary Video 2

Supplementary Video 3

## Figures and Tables

**Figure 1:**
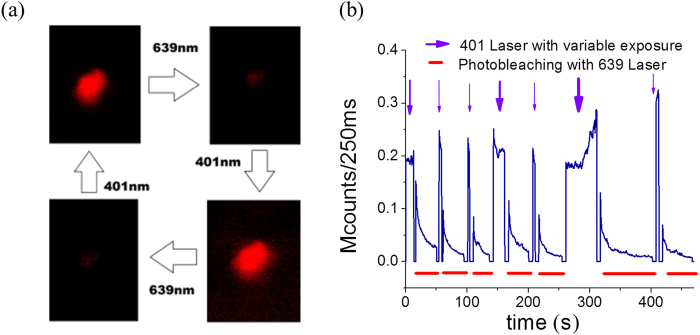
Photoswitching of carbon dots (**a**) The on-off switching observed by alternating 401 nm and 639 nm laser excitation. (**b**) Photon counts in a hybrid photomultiplier detector show photo-decay and subsequent gain in intensity (photoswitch) using 401 laser. The blue arrow shows the 401 nm pulses (detected in channel 1) and the red bars show the photo-decay with 639 nm Laser (detected in channel 2). The 4th and 6th cycle, with a slightly higher exposure time of blue light, results a better recovery of fluorescence.

**Figure 2:**
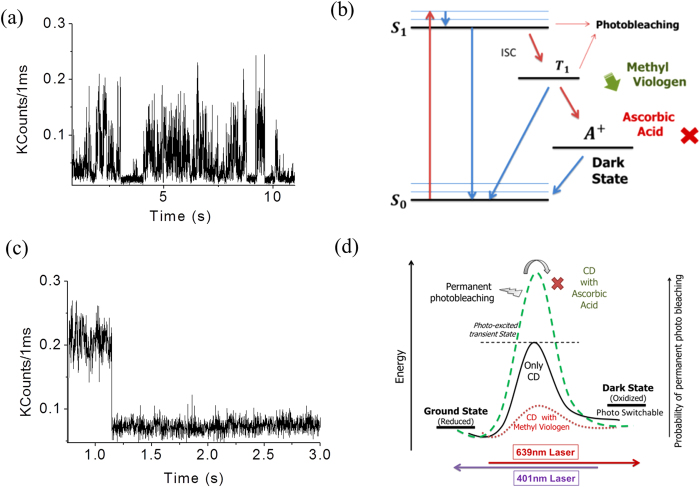
Single molecule time trace showing (**a**) radical blinking in presence of an electron acceptor (methyl viologen) and (**c**) single step bleaching in presence of an electron donor (ascorbic acid). (**b**) A Jablonski diagram and (**d**) an energy barrier diagram of the favored dark state formation in the red emissive carbon dots. Chemically lowering the energy barrier by an electron acceptor molecule favors the photoswitching, while an electron donor molecule retards the process.

**Figure 3:**
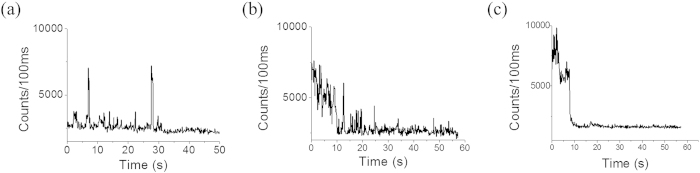
Characteristic single molecule time traces as recorded by EMCCD detection unit. Figure (**a**) and (**c**) represents the fluorescence trace in presence of 5 mM methyl viologen and 5 mM ascorbic acid respectively. (**b**) represents fluorescence trace without any electron donor/acceptor. Brightness decreases in presence of methyl viologen and increases in the presence of ascorbic acid.

**Figure 4:**
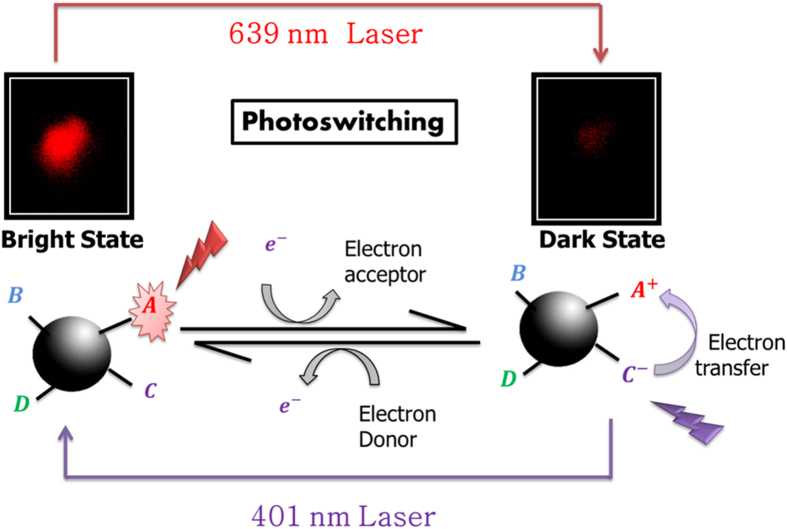
Electron transfer from the neighboring high energy traps can efficiently switch the chromophore back from the dark radical state to a bright fluorescent state. The high energy non-emissive traps which can effectively absorb the higher frequency of light might have a role at the core of photoswitching process.

**Figure 5:**
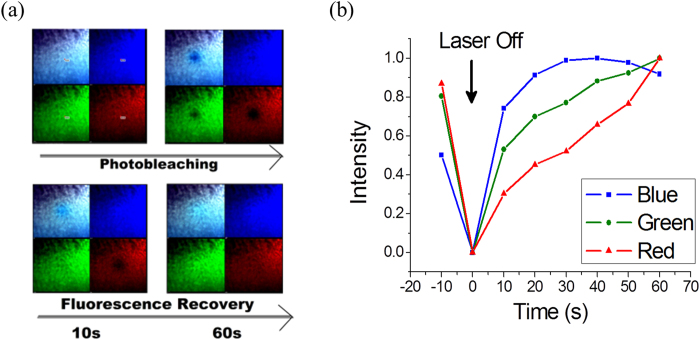
Fluorescence recovery after photobleaching within a self-assembled monolayer of carbon dots. (**a**) Three different lasers (401, 488 and 639 nm) were focused on a point region of interest to study comparative photobleaching. (**b**) The blue emission decays slowly and recovers fast, while the red emission decays fast and recovers slowly.
