# Supplementary material for: Reversible Photoswitching of Carbon Dots
Source: Sci Rep. 2015 Jun 16;5:11423. doi: 10.1038/srep11423 (PMC4468586; doi:10.1038/srep11423)
Supplement: Supplementary Information [file srep11423-s1.pdf]

Supplementary Information

Reversible Photoswitching of Carbon Dots

Syamantak Khan, Navneet Chandra Verma, Abhishek Gupta and Chayan Kanti Nandi*

School of Basic Sciences, Indian Institute of Technology Mandi, Himachal Pradesh, 175001, India.

Corresponding author

*Chayan K. Nandi

School of Basic Sciences,

Indian Institute of Technology Mandi,

Himachal Pradesh-175001, India

Email: chayan@iitmandi.ac.in

Tel No. 01905300047

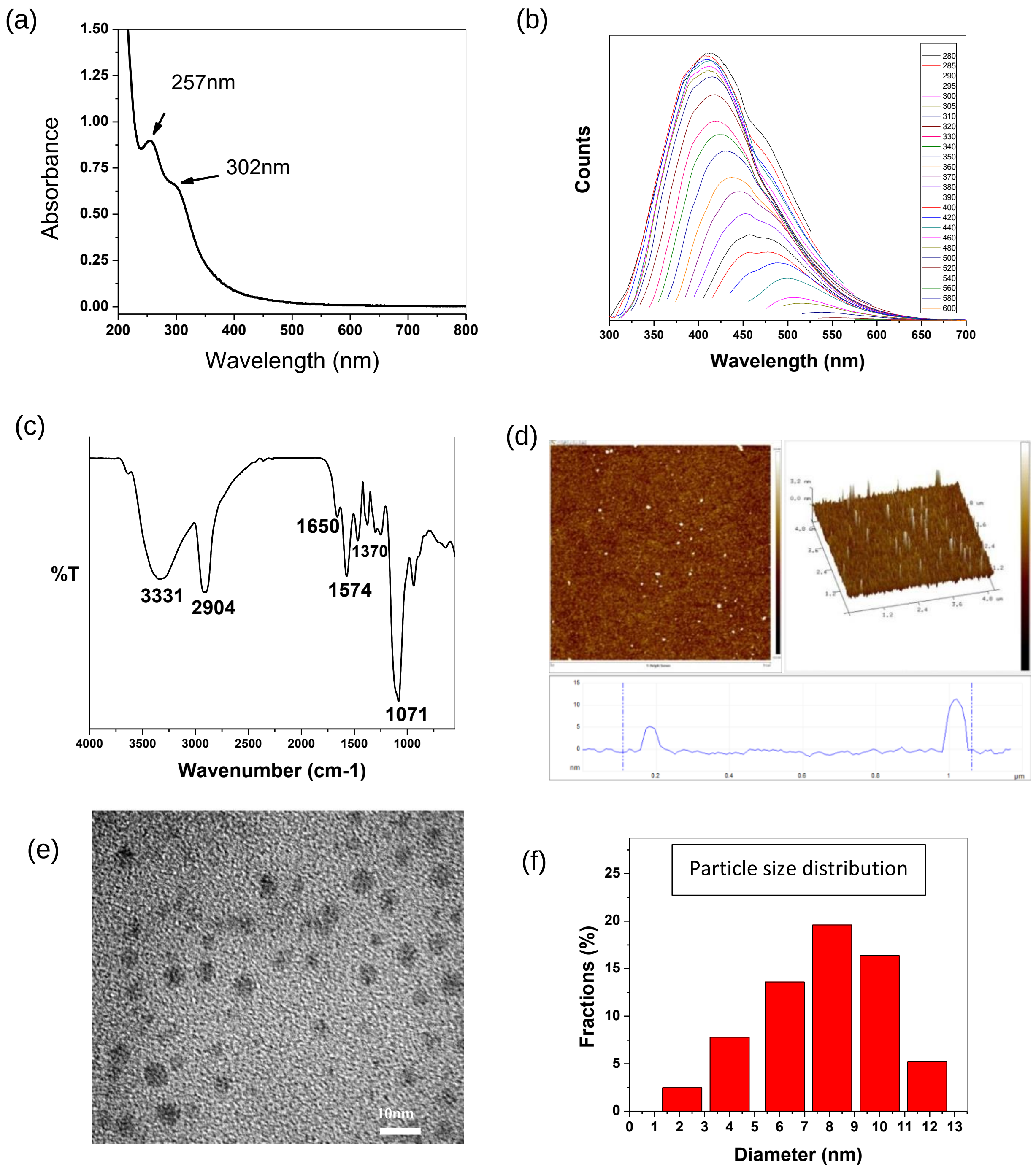

Figure S1: (a) UV Vis Absorbance spectra of synthesized carbon dot solution. (b) Ensemble fluorescence from carbon dots. The fluorescence spectrum is characterized by the typical multi-coloured fluorescence, i.e. shift of the emission maxima from shorter to longer wavelength with a change in excitation wavelength. (c) FTIR spectra of the synthesized carbon dots. Stretching vibrations at 1650 and 1574 cm^{-1} represents the C=O and N-H functional groups respectively. Specific C-O single bond stretching is shown at 1071 cm^{-1}. Broadening of peak at 3331cm^{-1} occurs due to presence of –OH groups. (d) Atomic force microscopic topography images of synthesized carbon dots are shown on silicon substrate. Lower panel shows the size distribution of particles through height profiling. (e, f) TEM images of synthesized carbon dots with particle size distribution.

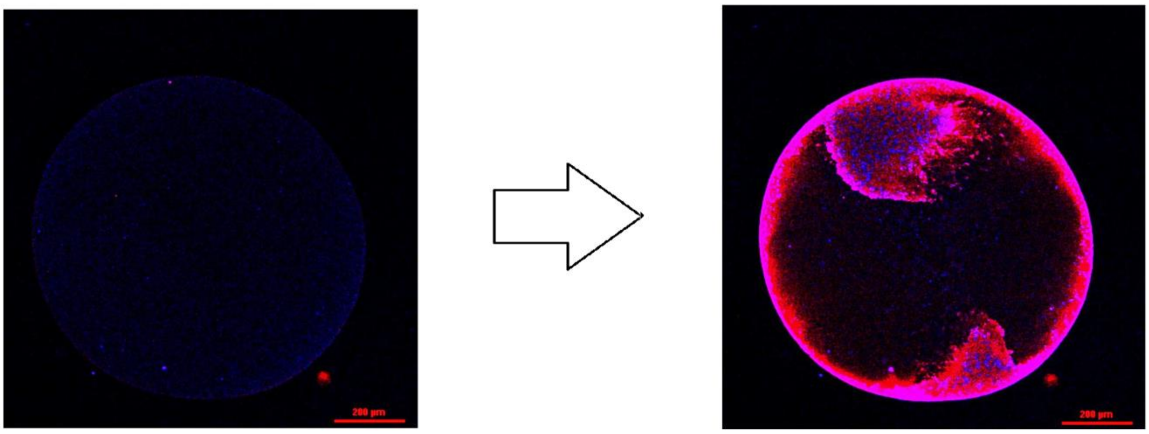

Figure S2. Intensity reversal of the carbon dots upon evaporation of the dropcasted solution. The intensity switch was observed from the blue emissive (dominant) liquid drop (left) to the enhanced red emissive (dominant) the dried spot (Right) with similar laser excitation.

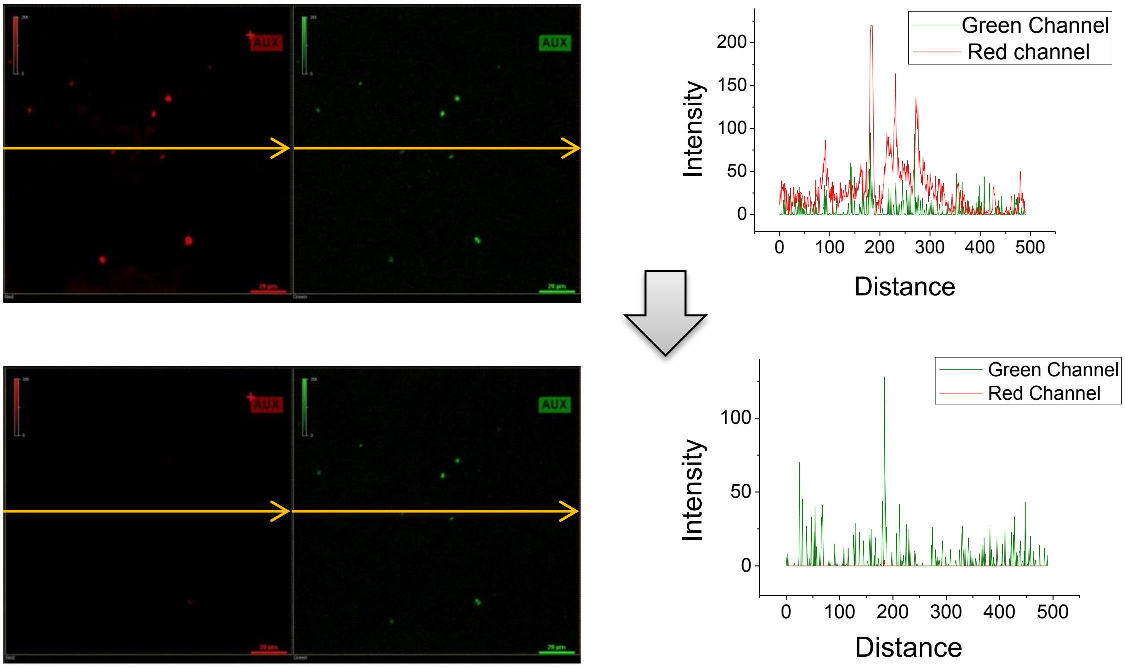

Figure S3. Fluorescence image of dried spot as observed in confocal microscope in two different channels. The red emission of the particles bleaches faster than the green emission. Intensity profiles show the change in intensity in two different channels along the marked yellow line. Upper panel shows the original intensity and the lower panel shows the intensity after photobleaching.

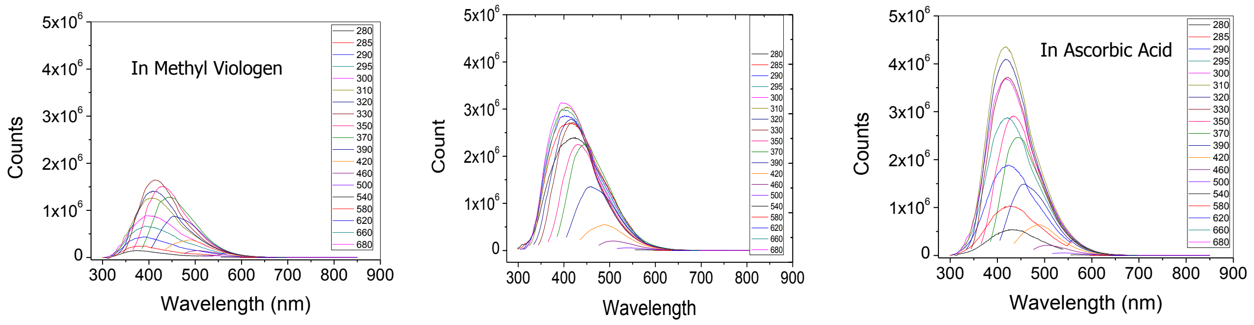

Figure S4. The role of chemical environment. The intensity of the fluorescence decreases in presence of methyl viologen but it increased in the presence of ascorbic acid.

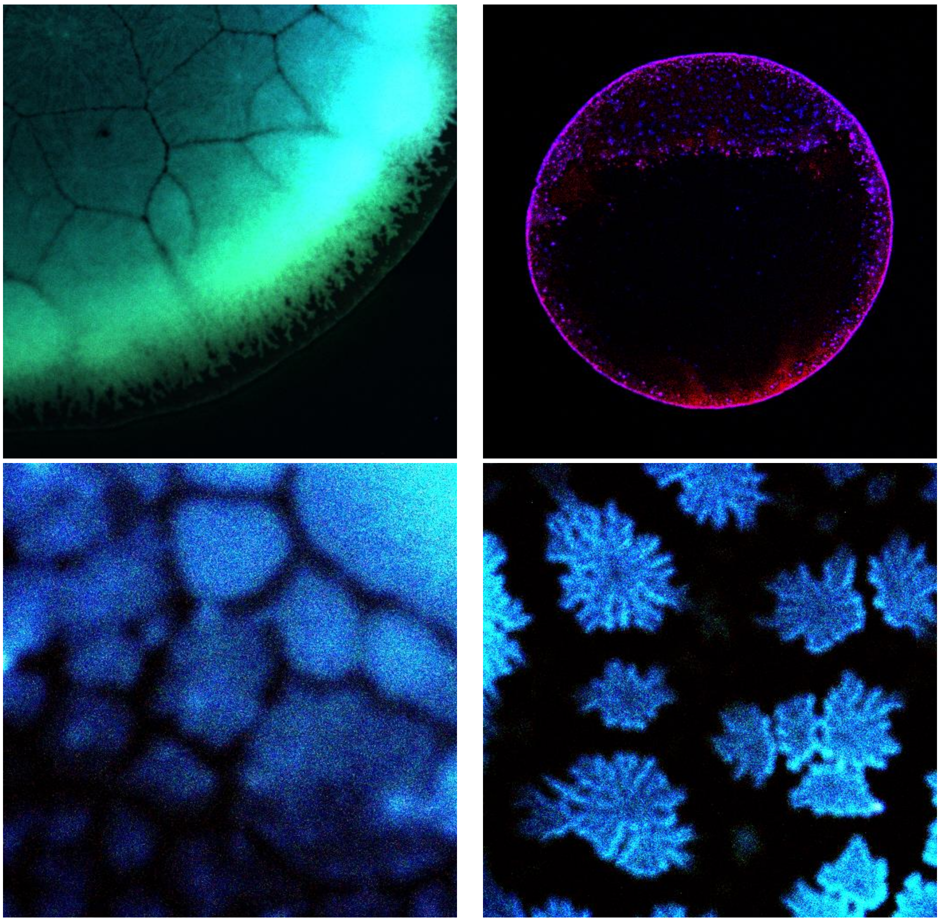

Figure S5. Concentration variation gives rise to different structures when carbon dots are evaporated. Higher concentrations results uniform patches, while a diluted solution produce a coffee ring pattern. Controlled experiments can be performed to obtain self-assembled nanoparticle colonies, possibly a monolayer of particles.

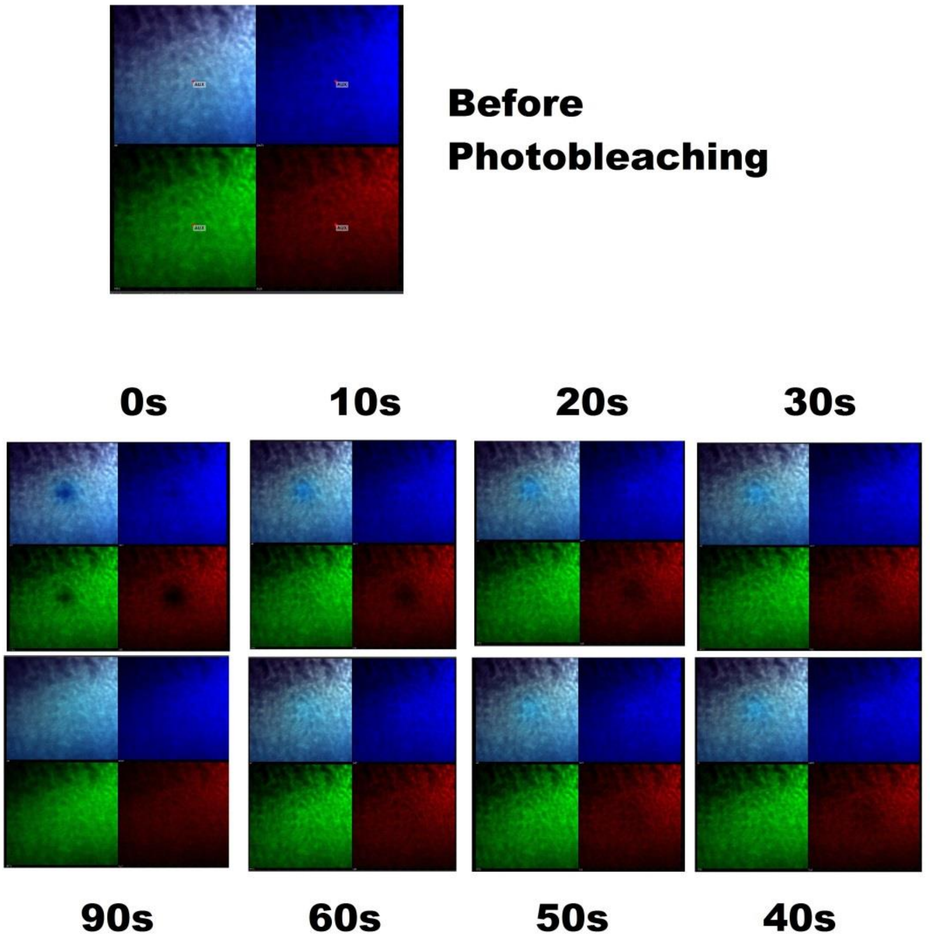

Figure S6. Fluorescence recovery after photobleaching in carbon dot monolayer. Images showing the intensity recovery at 10th,20th,30th,40th,50th,60th and 90th second. The recovery is practically complete before 1minute time.
